# Rotavirus VP6 protein mucosally delivered by cell wall-derived particles from *Lactococcus lactis* induces protection against infection in a murine model

**DOI:** 10.1371/journal.pone.0203700

**Published:** 2018-09-07

**Authors:** C. Facundo Temprana, Marcelo H. Argüelles, Nicolás M. Gutierrez, Patricia A. Barril, Laura E. Esteban, Dalila Silvestre, Marcelo G. Mandile, Graciela Glikmann, Alejandro A. Castello

**Affiliations:** 1 Laboratorio de Inmunología y Virología (LIV), Departamento de Ciencia y Tecnología, Universidad Nacional de Quilmes, Bernal, Buenos Aires, Argentina; 2 Consejo Nacional de Investigaciones Científicas y Técnicas (CONICET) Buenos Aires, Argentina; 3 Laboratorio de Microbiología de los Alimentos, Centro de Investigación y Asistencia Técnica a la Industria (CIATI A.C.)–CONICET, Centenario, Neuquén, Argentina; 4 Instituto de Ciencias de la Salud, Universidad Nacional Arturo Jauretche, Florencio Varela, Buenos Aires, Argentina; Instituto Butantan, BRAZIL

## Abstract

Rotaviruses are the primary cause of acute gastroenteritis in children worldwide. Although the implementation of live attenuated vaccines has reduced the number of rotavirus-associated deaths, variance in their effectiveness has been reported in different countries. This fact, among other concerns, leads to continuous efforts for the development of new generation of vaccines against rotavirus.In this work, we describe the obtention of cell wall-derived particles from a recombinant *Lactococcus lactis* expressing a cell wall-anchored version of the rotavirus VP6 protein. After confirming by SDS-PAGE, Western blot, flow cytometry and electronic immunomicroscopy that these particles were carrying the VP6 protein, their immunogenic potential was evaluated in adult BALB/c mice. For that, mucosal immunizations (oral or intranasal), with or without the dmLT [(double mutant Escherichia coli heat labile toxin LT(R192G/L211A)] adjuvant were performed. The results showed that these cell wall-derived particles were able to generate anti-rotavirus IgG and IgA antibodies only when administered intranasally, whether the adjuvant was present or not. However, the presence of dmLT was necessary to confer protection against rotavirus infection, which was evidenced by a 79.5 percent viral shedding reduction.In summary, this work describes the production of cell wall-derived particles which were able to induce a protective immune response after intranasal immunization. Further studies are needed to characterize the immune response elicited by these particles as well as to determine their potential as an alternative to the use of live *L*. *lactis* for mucosal antigen delivery.

## Introduction

Rotavirus (RV) has been described as the primary cause of acute gastroenteritis in children worldwide with an incidence that does not correlate to the socioeconomic characteristics of the population, although the mortality rates do [1]. Fortunately, since 2006 licensed vaccines against RV have been implemented [2,3]. Subsequently, these vaccines were introduced each year in different countries and global mortality rates in children below five years old were reduced from 528,000 in the year 2000 to 215,000 in 2013 [4]. The most used vaccines, whether Rotarix^®^ (GlaxoSmithKline Biologicals, Rixensart, Belgium) or RotaTeq^®^ (Merck & Co. Inc., West Point, PA, USA), consist of attenuated strains (human attenuated or human/bovine reassortant, respectively). They do not confer sterilizing immunity to the vaccinated subject but confer protection from severe diarrhea, thus preventing patient death mainly in those countries where adequate health care cannot be provided immediately [2,5].

Up to date, the vaccine performance does not seem to be a discussable issue, and the WHO recommends vaccination against rotavirus as an excellent strategy to reduce childhood death due to severe diarrhea [6]. However, some aspects of these vaccines have been questioned. One of them is the increased risk of intussusception observed with the first licensed RV vaccine Rotashield^®^, which was taken off the U.S market because of this cause [7]. This problem has not been reported as a considerable side effect for the currently licensed vaccines since it resulted in being five to ten times lower than that found for Rotashield^®^, and the benefits of vaccination far exceed the risk for this pathology [6]. Another issue is related to the fact that the vaccine strains replicate in the vaccinated person who sheds typically large amounts of these viruses to the environment. This viral shedding is more intense and can last for several weeks or months if severely immunocompromised patients are infected or inadvertently inoculated [8]. In this way, viable vaccine strains currently being administered massively can be relatively abundant in the environment making possible the emergence of reassortants with human and animal wild strains. In fact, evidence has been presented involving vaccine and wild-type RV reassortants strains in severe human cases [9–12]. Hence, oral RV vaccine strains can persist in the environment and significantly influence RV ecology [13]. Moreover, as current oral vaccines do not induce sterilizing immunity, continuous circulation of RV, along with the selective driving force introduced by vaccination, can lead to the emergence of new pathogenic strains that might not be covered by the vaccine in the future. No evidence has been produced yet indicating that RV vaccination can lead to antigenic drift in surface proteins of the prevalent strains [9,14–16]. However, totally G and P heterotypic and other genomic differences can introduce an evolutive bias toward vaccine immunity resistant strains [17,18]. Early evidence of this could be the observation that the G1P[8] genotype (dominant human strains in the pre-vaccine era), is relatively less abundant after vaccine introduction in Latin American countries and some evidence that DS-1 strains are concomitantly favored [13,19]. On the other hand, in less developed settings strain diversity and mainly host factors appear to be interfering with the effectiveness of oral vaccines [20].

For the above exposed, a new generation of RV immunogens that do not include a living microorganism and that induce stronger and wider immunity is desirable. In 1999 Ward´s group [21] proposed the use of VP6 protein (the component of the middle layer of the triple-layered RV viral particle) as the only rotavirus antigen being a potential candidate for an RV subunit vaccine. The immune response against this protein has been shown to confer protection against infection in a mouse model although it does not induce neutralizing antibodies. Since VP6 is highly conserved within the same RV group, it can confer heterotypic protection [21,22]. The use of VP6 as a possible candidate vaccine as well as the plausible mechanisms behind the VP6-induced protection have been extensively reviewed by Ward et al., 2010 and Jalilvand et al., 2015 [23,24].

*Lactococcus lactis* has been proposed as a promising candidate for the delivery of therapeutic/prophylactic molecules to the gastrointestinal mucosa, mainly for its “generally recognized as safe” (GRAS) status [25]. Different expression systems were developed for this bacterium, being the nisin-controlled gene expression (NICE) system the most commonly used [26,27]. In the last decade, numerous studies have been performed exploring *L*. *lactis* as a delivery platform to mucosa for a wide variety of antigens involving alternative strategies of antigen localization (in the cytoplasm, cell wall-anchored or secreted to the extracellular space) [27,28]. Also, as an alternative to the usage of the living bacteria, the use of gram-positive enhancer matrix (GEM), also known as bacterium-like particles (BLP) derived from *L*. *lactis* has been proposed [29,30]. Rotaviral proteins NSP4, VP8, VP7, and VP4, have been expressed in *L*. *lactis* [31–36] and our group expressed VP6 protein in a cell wall-anchored version in 2013 [37]. Although the immunogenicity or capacity to induce neutralizing antibodies against RV was evaluated, none of the previous studies assayed their potential to prevent/reduce viral infection in the murine model.

In this work, we propose the cell wall-derived particles (CWDP) as an alternative to the use of living *L*. *lactis* or the BLP system. We describe for the first time their obtention from a recombinant *L*. *lactis* expressing a cell wall-anchored RV VP6 protein, their characterization, and their immunological evaluation. Both, the immune response generated after intranasal and intragastric inoculation of these CWDP, with or without dmLT [(double mutant *Escherichia coli* heat labile toxin LT(R192G/L211A)] adjuvant and the ability to confer protection against RV infection in the murine model were evaluated.

## Material and methods

### Expression of VP6 protein in *L*. *lactis*

The generation of *L*. *lactis* expressing RV VP6 protein and protein expression optimization was previously described by Esteban et al. [37]. Briefly, *L*. *lactis* carrying VP6 protein or control *L*. *lactis* NZ9000 (strain containing no plasmid) were grown in M17 broth (Biokar Diagnostics, Beauvais, France) containing 0.5% w/v of sucrose (GM17) with or without chloramphenicol 10 μg/ml, respectively. Bacteria were cultured overnight (ON) at 30°C without shaking. Then, a 1:20 dilution was performed in fresh medium and when an optical density at 600 nm (OD_600_) of 0.5 was reached, nisin (Danisco, Grindsted, Denmark) was added to a final concentration of 100 ng/ml to induce protein expression. Induced cultures were incubated for two hours at 30°C before processing [37].

### Generation of CWDP

Induced bacteria cells from an 800 ml culture were concentrated by centrifugation at 3840 xg in a J-12 Beckmann centrifuge. The pellet was washed twice with SM buffer (Tris 50 mM, NaCl 100 mM, Mg_2_SO_4_ 10 mM; pH 7.5) [38] and resuspended in 25 ml of the same buffer. The bacterial suspension was passed five times through a French Press (Thermo Fisher Scientific, Waltham, MA, USA) at 15,000 psi to induce cell wall disruption. Then, unbroken cells were pelleted with a 3840 x g centrifugation at 4°C. The supernatant containing cell wall fragments was recovered and centrifuged for 15 min at 7520 x g at 4°C. The pellet was washed and resuspended in 400 μl of SM buffer and kept at -20°C until used. This concentrated fraction of broken cell walls will be referred to in this paper as CWDP.

### Characterization of CWDP

#### SDS-PAGE, Western blot, and flow cytometry

To confirm the presence of VP6 protein in the CWDP obtained from the recombinant *L*. *lactis*, SDS- PAGE, Western blot, and ELISA were performed.

Briefly, for SDS-PAGE 10% analysis, 20 μl of CWDP sample was mixed with 58 μl of distilled water, 2 μl of SDS 20% w/v and 20 μl of PAGE loading buffer and incubated at 100°C for 10 min. After electrophoresis at 100 V for 2.5 h, gels were Coomassie-blue stained or blotted onto a PVDF membrane. Protein Molecular Weight Marker (MWM), PageRuler™ Prestained Protein Ladder, 10 to 180 kDa (Thermo Fisher Scientific) was included for size comparison.

The membrane was incubated ON at 4°C with PBS containing 3% w/v casein. Then, the membrane was incubated with a 1/3000 dilution in PBSTC (PBS, 0.2% v/v Tween-20 and 1% w/v casein) of an in-house produced mouse polyclonal antibody anti-RRV rotavirus strain for 1 h at 37°C. After that, the membrane was washed three times with PBST (PBSTC without casein) and incubated for 1 h at 37°C with a 1/1000 dilution of HRP-conjugated goat anti-mouse IgG (Pierce Biotechnology, Rockford, IL, USA). Antibody detection was performed with a chemiluminescent substrate (PBL, Bernal, Buenos Aires, Argentina) following the manufacturer’s instructions and the x-ray film was exposed until bands were visualized.

To further confirm the presence of VP6 protein, the CWDP obtained from induced *L*. *lactis* NZ9000 (control) and recombinant *L*. *lactis* expressing VP6 protein were analyzed by flow cytometry. For this, CWDP were incubated for 30 min at 37°C with a 1/2000 dilution in PBSTC of an in-house produced rabbit polyclonal antibody anti-RRV rotavirus. After three washes with PBST, CWDP were incubated for 30 min at 37°C with a 1/1000 dilution in PBSTC of a FITC-conjugated goat anti-rabbit IgG antibody (Chemicon, Temecula, CA, USA). Then, two washes with PBST were performed, and CWDP were resuspended in PBS buffer for flow cytometry analysis. A gate including CWDP was selected on forward scatter and side scatter dot plots, and 100,000 events were acquired within this gate in a FACScalibur flow cytometer (Becton Dickinson, Immunocytometry Systems, San Jose, CA, USA). CWDP from *L*. *lactis* NZ9000 treated as previously described were used to set negative values of fluorescence in FL1-H channel with a band-pass filter of 530 nm (515 to 545 nm).

#### Light scattering

CWDP size was analyzed by dynamic light scattering (DLS) using a Zetasizer Nano (Malvern Instruments, Malvern, UK).

#### Transmission electron microscopy and immunogold staining

CWDP samples were placed on collodion-coated copper grids (400 mesh) and stained with 0.5% w/v phosphotungstic acid for 5 seconds. The grids were examined using a JEM 1200 EX II (JEOL Ltd., Tokio, Japan) transmission electron microscope and pictures taken with an Erlangshen ES1000W, model 785 (Gatan Inc., Pleasanton, CA, USA) camera at SCME Facultad de Ciencias Veterinarias, UNLP, La Plata, Argentina.

For immunostaining CWDP were incubated for 30 min at 37°C with a 1/500 dilution in PBSTC of a rabbit polyclonal antibody anti-RRV rotavirus. After three washes with PBST, CWDP were incubated for 30 min at 37°C with a 1/5 dilution in PBSTC of a goat anti-rabbit IgG conjugated to 10 nm gold particles antibody (Sigma-Aldrich, St. Louis, MO, USA). Then, two washes with PBST were performed, and CWDP were resuspended in PBS buffer for further microscopy analysis.

### Mice immunization and sample collection

Adult male BALB/c mice (25 per assay) were obtained from the Bioterio de la Facultad de Ciencias Veterinarias de la Universidad Nacional de La Plata (FCV-UNLP). During experiments, the animals were kept in the Bioterio at the Universidad Nacional de Quilmes (5 animals/cage), with a 12 h light/ 12 h dark cycle at controlled temperature (21 ± 1°C). Water and commercial rodent food were administered *ad libitum*. Mice were monitored during the experiment three times a week for their overall health status evaluating indicators like activity, normal consumption of food and water, interaction with cage mates, and general appearance.

All procedures were performed to minimize animal suffering, and at the end of experiments, animals were sacrificed by a ketamine/ xylazine overdose.

Groups of 5 BALB/c mice (6 to 8 weeks old) were immunized intranasally or intragastrically via gavage (using a 22G-ball tip needle) with CWDP containing 30 μg of VP6 per dose as determined by ImageJ densitometry analysis performed on a Coomassie-blue stained SDS-PAGE using BSA as mass standard. Intranasal immunization was performed on days 0, 14 and 28; and for intragastric administration, three series of three doses each were used on days 0, 1, 2; days 14, 15, 16 and days 28, 29 and 30. As a mucosal adjuvant, 10 μg of dmLT was used in doses corresponding to days 0, 14 and 28 in both administration routes. A group immunized with PBS was included as a control. Mice sera were collected on days 0, 14, 28 and 42 and kept at -20°C until used. All procedures involving animals were approved by the IACUC of the Universidad Nacional de Quilmes (Approved Protocol N° 003/16).

### Detection of RV specific IgA and IgG antibodies by ELISA

Detection of specific antibodies against RV was performed by ELISA. For this, 96-well plates were coated with concentrated RV (RRV strain) in carbonate buffer pH 9.0, ON at 4°C. Sera were tested in a 1/100 or 1/20 dilution for detection of IgG or IgA, respectively, and IgG positive sera were titered using threefold dilution series in PBSTC. Plates were incubated for 1 h with a 1/1500 dilution in PBSTC of HRP-conjugated goat anti-mouse IgG (Fc) antibody (Pierce Biotechnology, Rockford, IL, USA). After washing three times with PBST, o-phenylenediamine peroxidase substrate was added, and after 15 min the reaction was stopped with sulfuric acid, and the optical density at 490 nm (OD_490_) was determined. For RV specific IgA determination in stools samples, 96-well plates were coated at room temperature for 1 h with a capture rabbit anti-mouse IgA antibody (Thermo Fisher Scientific) diluted 1/200 in carbonate buffer. Then, 10% w/v stool samples prepared in TNC buffer (Tris 10 mM, NaCl 140 mM, CaCl_2_ 5 mM) with 0.05% v/v Tween 20 (TNCT) were diluted 1/5 in PBSTC and incubated for 1 h at 37°C. After that, concentrated RV (RRV strain) was added and incubated ON at 4°C. Purified biotinylated goat IgG anti-RRV RV was added in a 1/500 dilution in PBSTC and incubated for 1 h at 37°C. Finally, streptavidin-HRP (USBiological, Salem, MA, USA) diluted 1/2000 in PBSTC was added and incubated for 30 min at 37°C. Then, the o-phenylenediamine substrate was added, and after 15 min the reaction was stopped with sulfuric acid, and the OD_490_ was determined. Each incubation step was performed in a humidity chamber and was followed by three washes with PBST.

### Virus challenge and determination of viral shedding

Immunized mice were infected with wild-type RV EC strain 43 days after the first immunization to evaluate RV shedding. For this, 10^4^ shedding doses (SD50) were administered intragastrically, and stool samples were collected for each mouse every day for eight days. Stools were stored at -80°C until all samples were collected, and then 10% w/v suspension were prepared in TNCT. After completely dissolving the fecal pellets, samples were centrifuged at 500 xg for 10 min to remove debris. Supernatants were used to determine the presence of RV antigens by ELISA as previously described [39]. RV antigens shedding curves were constructed plotting the OD_490_ versus days post-challenge for each mouse within each group. Then, the area under the curve (AUC) was calculated for each animal and compared to that of the PBS immunized group considered as 100% viral shedding [40].

### Statistics

Statistics were performed with GraphPad Prism software (La Jolla, CA, USA) and the results are expressed as mean ± SE. The statistical tests used are mentioned in the figure captions and results were considered to be significantly different when *P*< 0.05.

## Results

### Generation and characterization of CWDP

**Fig 1:**
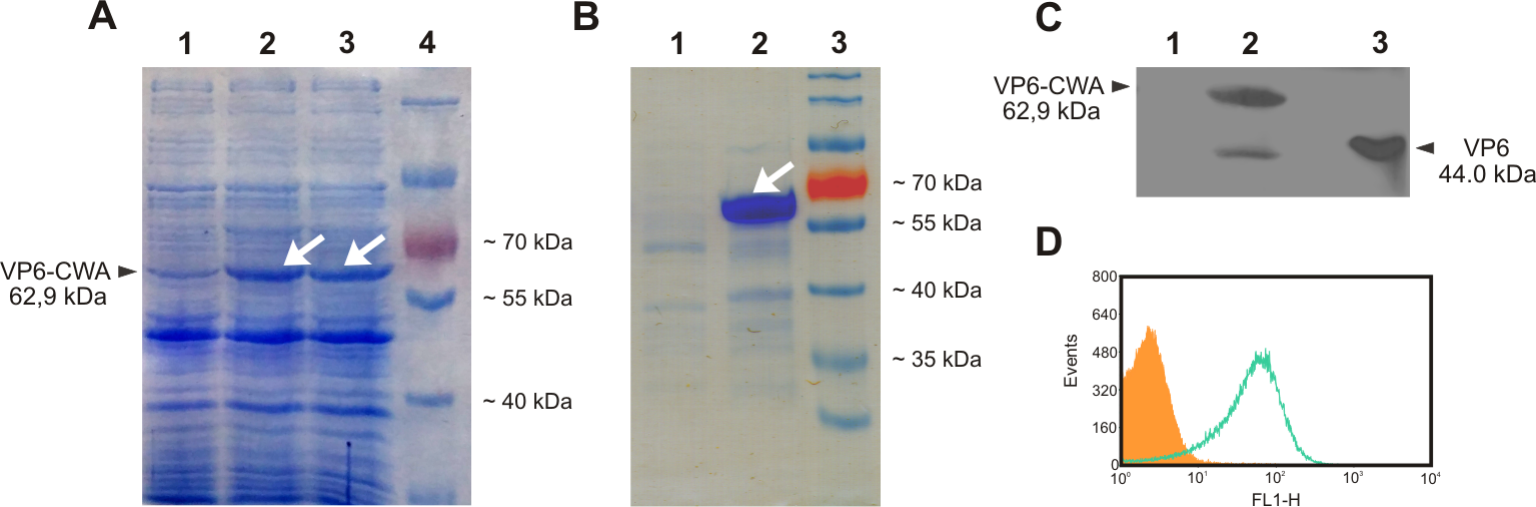
Analysis of the protein content of the CWDP. **A—**Coomassie-blue stained SDS-PAGE corresponding to the total protein extract from: **lane 1**, non-induced *L*. *lactis* carrying RV VP6 protein; **lanes 2 and 3**, nisin-induced *L*. *lactis* expressing RV VP6 protein; **lane 4**, protein Molecular Weight Marker. **B—**Coomassie-blue stained SDS-PAGE corresponding to the CWDP obtained from: **lane 1**, untransformed *L*. *lactis* NZ9000 (NZ-CWDP); **lane 2**, recombinant *L*. *lactis* expressing RV VP6 protein (VP6-CWDP); **lane 3**, protein Molecular Weight Marker. **C—**Western blot analysis of the CWDP obtained from: **lane 1**, untransformed *L*. *lactis* NZ9000 (NZ-CWDP); **lane 2**, recombinant *L*. *lactis* expressing RV VP6 protein (VP6-CWDP); **lane 3**, positive control of RV particles. **D—**Histogram plot corresponding to flow cytometry analysis of CWDP obtained from untransformed *L*. *lactis* NZ9000, used to set negative FITC fluorescence (filled orange curve) or recombinant *L*. *lactis* expressing RV VP6 protein (unfilled green curve). VP6 protein was stained with a polyclonal anti-RV serum and a FITC-conjugated secondary antibody. Protein molecular weight markers (MWM) sizes as well as expected sizes of VP6 and VP6 fusion to cell wall anchor domain (VP6-CWA) bands are depicted in kiloDaltons (kDa). White arrows indicate bands corresponding to VP6-CWA protein.

In previous work, the generation of a recombinant *L*. *lactis* expressing RV VP6 under the control of the nisin-inducible PnisA promotor, and anchored on the bacterial cell wall, was described [37]. This bacteria and *L*. *lactis* NZ9000 (same *L*. *lactis* strain without the construction carrying the VP6 gene) were used to generate the CWDP. Fig 1A and 1B show Coomassie-blue stained SDS-PAGE from total bacterial cell lysates and the CWDP, respectively. As it can be clearly seen from the comparison of Fig 1A and 1B, the CWDP preparation did not only result in the expected host’s protein amount reduction but also in an increased concentration of the VP6 protein.

The Western blot performed with polyclonal anti-RV antibodies revealed a band with the expected mass weight of 62.9 kDa, confirming protein identity (Fig 1C). Furthermore, to demonstrate VP6 presence within the CWDP, flow cytometry was used. As it can be seen in the histogram presented in Fig 1D the fluorescence values registered in FL-1 channel for FITC-conjugated secondary antibody used to detect the polyclonal anti-RV, shifted to higher values when compared to those obtained for CWDP derived from control *L*. *lactis* NZ9000 following the same staining protocol.

**Fig 2:**
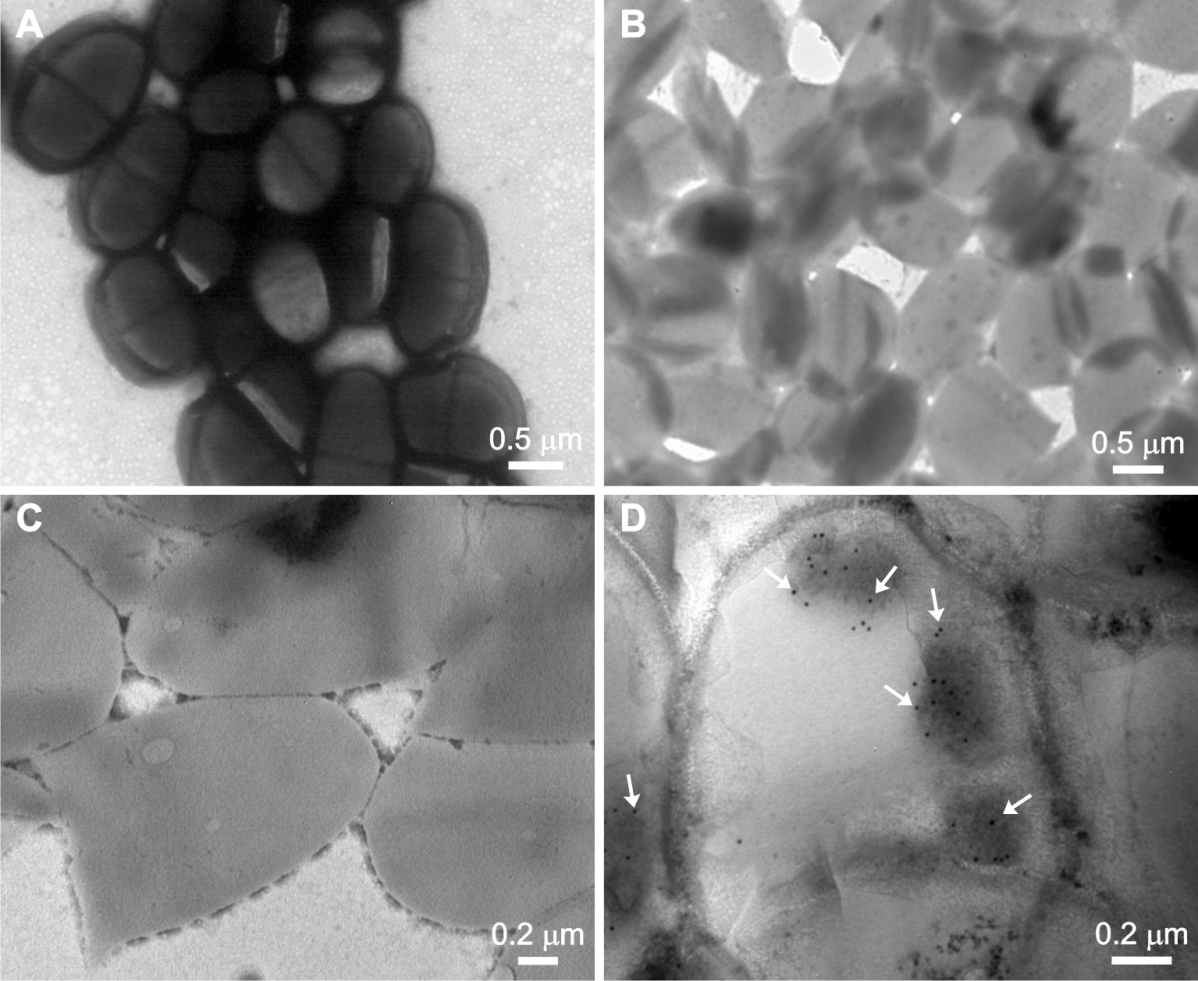
CWDP structure analysis by TEM. **A—**Entire recombinant *L*. *lactis* at 20,000 X magnification. **B—**CWDP obtained from recombinant *L*. *lactis* expressing RV VP6 protein at 20,000 X magnification. **C—**CWDP obtained from recombinant *L*. *lactis* expressing RV VP6 protein at 40,000 X magnification. **D—**CWDP obtained from recombinant *L*. *lactis* expressing RV VP6 protein at 60,000 X magnification. VP6 protein was detected with a polyclonal anti-RV serum and a secondary antibody labeled with 10 nm colloidal gold. Arrows indicate the electron-dense gold particles which are indicative of VP6 protein presence.

To gain insight into CWDP structure transmission electron microscopy was performed. Fig 2A shows the obtained images corresponding to the entire *L*. *lactis* and the CWDP (Fig 2B to 2D). According to the size estimation from the picture, the resulting CWDP have a size of approximately 1 μm. This value is lower than that obtained with the light scattering measurements, giving a mean size of 1901.8 nm with a polydispersity of 0.4792. Moreover, RV VP6 presence within the CWDP was also confirmed by immunogold electron microscopy (Fig 2D).

### Detection of RV specific antibodies

Animal immunization was performed with CWDP obtained from *L*. *lactis* expressing VP6 protein administered intranasally or intragastrically. The presence of specific IgG or IgA against rotavirus was evaluated in the mice sera collected on day 42 after the first immunization dose.

**Fig 3:**
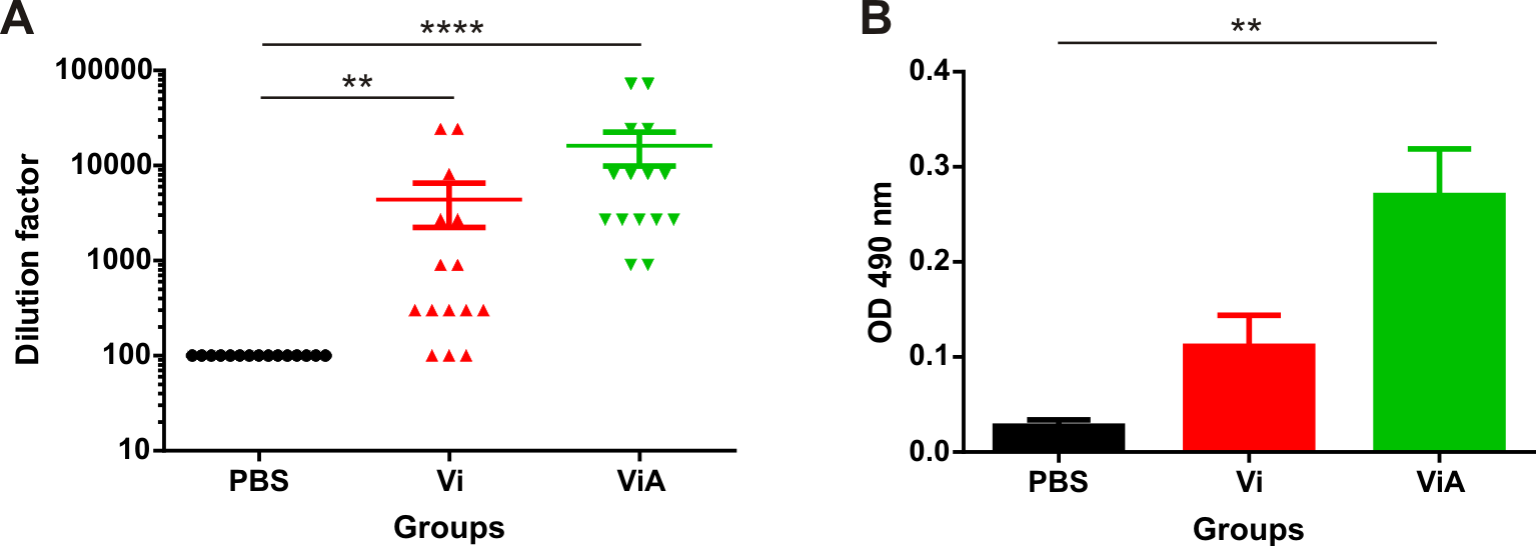
Anti-rotavirus IgG and IgA detection in serum at day 42 after the first immunization doseot. y with or without dmLT presented both IgG and IgA specific antibodies against RV. described in th. **A—**Anti-rotavirus IgG titer determined for each mouse in three independent assays and **B—**OD_490 nm_ obtained for a 1/20 dilution of each mouse serum in the IgA determination ELISA. Vi and ViA denote groups administered intranasally with VP6-CWDP without or with dmLT, respectively. Lines or bars represent the mean and error bars represent standard error for each group. Statistical analysis was performed by Kruskal-Wallis test using Dunn’s Multiple Comparison post-test. **<0.01, ****< 0.0001.

The groups that were administered CWDP intranasally with or without dmLT presented ELISA detectable IgG and IgA specific antibodies against RV (Fig 3), meanwhile, no specific anti RV antibodies were found in any mice immunized with CWDP intragastrically with or without dmLT.

Although specific anti-RV IgG and IgA were detected in sera at day 42 in groups immunized intranasally with VP6-CWDP, no specific anti-RV IgA in the feces collected at day 42 (before viral challenge) was detected by ELISA in any of the assayed groups.

### Protection against RV infection

**Fig 4:**
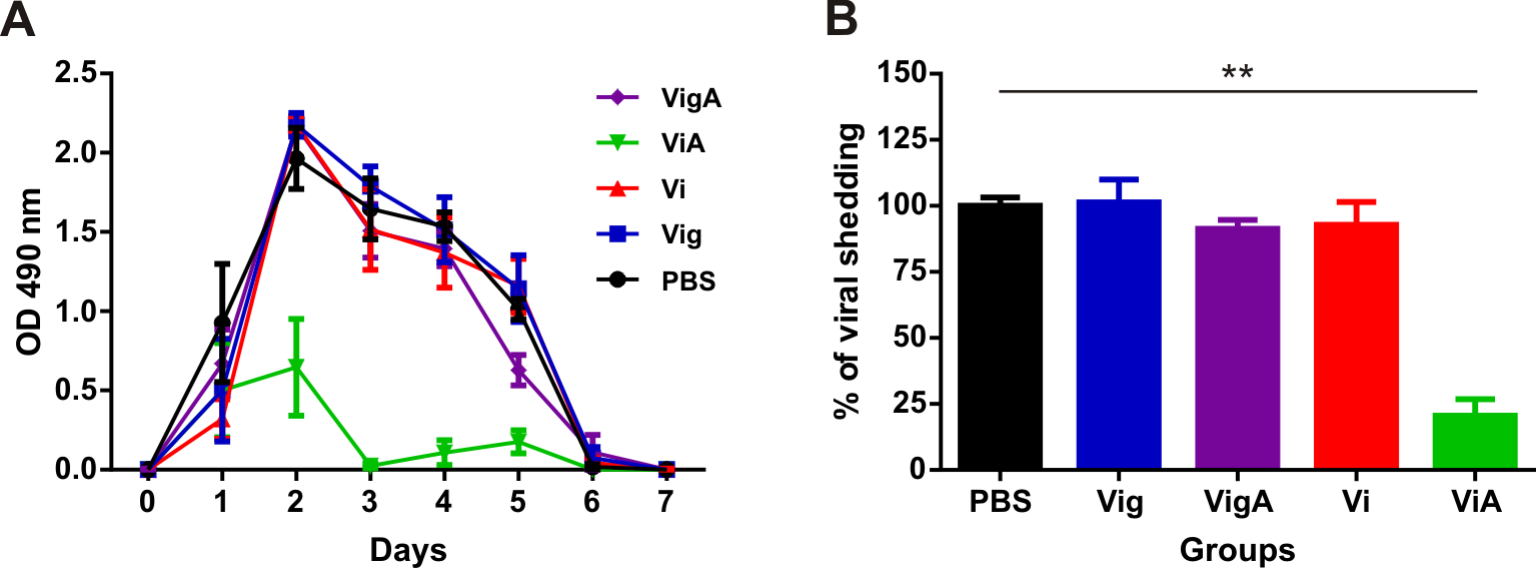
Rotavirus challengeot. y with or without dmLT presented both IgG and IgA specific antibodies against RV. described in th. **A—**Mean shedding curves obtained for mice groups immunized with PBS, VP6-CWDP intranasally (**Vi**), VP6-CWDP intragastrically (**Vig**), VP6-CWDP + dmLT intranasally (**ViA**) or VP6-CWDP + dmLT intragastrically (**VigA**). Each symbol represents the mean, and the error bars represent the standard error for each day. **B—**Percentage of viral shedding for each mouse within different groups respect to the 100% calculated for PBS. Bars represent the mean and error bars represent the standard error for each group immunized with: **PBS**, VP6-CWDP intranasally (**Vi**), VP6-CWDP intragastrically (**Vig**), VP6-CWDP + dmLT intranasally (**ViA**), VP6-CWDP + dmLT intragastrically (**VigA**). Statistical analysis was performed by Kruskal-Wallis test using Dunn’s Multiple Comparison post-test. **<0.01.

Every mouse from the previous experiment was challenged 43 days after the first immunization dose. The presence of rotaviral antigens in the stools was determined by ELISA for each mouse within each group every day for eight days. Results were plotted as the OD_490 nm_ obtained in the ELISA for each day, and the AUC was calculated for each mice. Fig 4A shows the mean shedding curve obtained for groups immunized with PBS, VP6-CWDP intranasally (Vi), VP6-CWDP intragastrically (Vig), VP6-CWDP + dmLT intranasally (ViA) or VP6-CWDP + dmLT intragastrically (VigA), respectively.

The mean AUC was calculated, and that corresponding to the PBS group was taken as the 100% of viral shedding. Fig 4B shows the percentage of viral shedding of each mouse calculated based on the mean obtained for PBS. The group intranasally immunized with VP6-CWDP and dmLT (ViA) presented a 79.5% reduction in rotavirus shedding when compared to that immunized with PBS.

**Fig 5:**
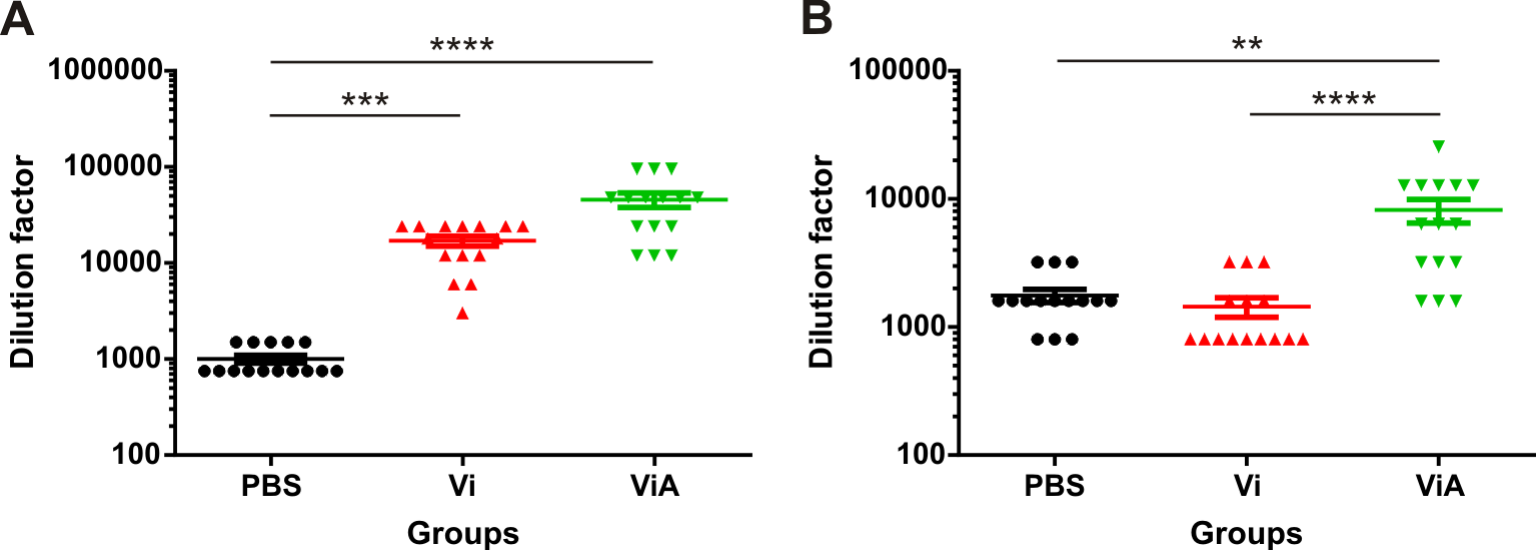
Anti-rotavirus IgG and IgA titration in serum at day 10 after infection with rotavirusot. y with or without dmLT presented both IgG and IgA specific antibodies against RV. described in th. **A—**Anti-rotavirus IgG titer determined for each mouse in three independent assays and **B—**Anti-rotavirus serum IgA titer determined for each mouse in three independent assays 10 days after infection with RV for groups administered intranasally with VP6-CWDP with or without dmLT (ViA or Vi, respectively) or PBS. Lines represent the mean and error bars represent the standard error for each group. Statistical analysis was performed by Kruskal-Wallis test using Dunn’s Multiple Comparison post-test. **<0.01, ***< 0.001, ****< 0.0001.

Titration of specific serum anti-rotavirus IgG and IgA was performed for those groups immunized intranasally with VP6-CWDP, with or without dmLT, and for those immunized with PBS 10 days post-infection with rotavirus (Fig 5).

## Discussion

In recent years the potential of *L*. *lactis* as live vaccine vectors has begun to be perceived. They are of great importance from the public health point of view since they have a high margin of safety with low production costs and are very easy to administer [41]. Different recombinant strains of *L*. *lactis* expressing a large number of antigens derived from bacteria, viruses, and parasites have been described as potential mucosal vaccines [26, 42–46].

The absence of an outer membrane in *L*. *lactis* makes it particularly attractive for the exposure of bioactive molecules on the bacterial surface. Thus, anchoring proteins or peptides to the cell wall has emerged as a promising application in several fields of biotechnology [47] and this strategy has been widely used for the evaluation of *L*. *lactis* as a delivery system for mucosal antigens [46]. However, sometimes the low amount of antigen available on the surface and concerns associated with the genetic modifications of *L*. *lactis* have driven the development of alternative strategies. One of them is the gram-positive enhancer matrix or GEM, also known as bacterium-like particles or BLP [29,30].

Briefly, in the case of live *L*. *lactis*, the expression and exposure of the heterologous protein occur in the same cell that contains the protein-CWA fusion gene in the expression plasmid or the genome. In contrast, the expression and binding of the fusion protein in the GEM strategy are separate processes. This requires an organism (bacteria, yeast, etc.) that expresses a recombinant fusion protein comprising the delivery subject antigen fused to a peptidoglycan-binding peptide and a purification system thereof. On the other hand, a wild-type *L*. *lactis* subjected to an acid and heat treatment generates bacterial death and the emptying of its cytoplasmic content giving rise to the GEM [48,49].

These particles are dead and deprived of intracellular content and surface proteins in their native version. However, the peptidoglycan layer of the cell wall remains intact and provides the structural rigidity to form particles that maintain the shape of the bacteria with a size of approximately one micrometer in diameter (GEM) [29]. Finally, incubation with the purified fusion antigen "decorates" the surface of the GEM allowing its vehiculization [29,50].

Different studies demonstrated that the antigens exposed on the GEM particles induce a higher immune response than the soluble antigen. Thus, it is speculated that GEM particles are a promising adjuvant for intranasal immunization [30,51–53]. There are several precedents for mucosal immunization using BLP derived from *L*. *lactis*, such as BLP bound to the LcrV protein of *Yersinia pestis*, which succeeded in inducing mucosal and systemic immunity in newborn mice and provided complete protection against infection [30]. Likewise, BLP technology has been successfully tested in adult mice against the respiratory syncytial virus [54], malaria [55] and *Streptococcus pneumoniae* [56].

However, this type of vaccination approach implies the need to include two separate procedures. One, associated with obtaining the GEM and the other, the production and purification of the immunogen fused to a peptidoglycan binding domain. Finally, the BLP is achieved with the immunogen associated to the GEM through molecular interactions and not through covalent bonds [49].

In this work, an alternative to the use of live *L*. *lactis* or BLP was investigated.

### Generation and characterization of CWDP

In this work, CWDP derived from recombinant *L*. *lactis* expressing the VP6 protein of rotavirus have been generated and characterized. To produce them, the same *L*. *lactis* that expresses a version anchored to the cell wall of the VP6 protein is subjected to a mechanical lysis process that allows cell rupture. The resulting membrane/wall fragments are washed and concentrated generating the stock of CWDP. This strategy not only allows reducing the proteins derived from *L*. *lactis* but also allows concentrating the expressed antigen while maintaining the particulate structure (Fig 2).

In this sense, poor immune responses reported by some groups using live *L*. *lactis* have been attributed to the low amount of the expressed antigen in this system [57].

Moreover, in our laboratory, it was not possible to induce a humoral immune response detectable by ELISA after intranasal or intragastric immunizations with live recombinant *L*. *lactis* (the same one used in this work to generate the CWDP). Since the immunogenicity of the VP6 expressed in the mentioned *L*. *lactis* has been previously demonstrated [37], it is conceivable that the low amount of antigen administered in a tolerogenic environment such as the mucosa could be responsible for the absence of the immune response.

To evaluate the immunogenicity of VP6 in the context of the CWDP mice were immunized intranasally and intragastrically with and without the addition of the mucosal adjuvant dmLT.

At day 42 after the first immunization, it was not possible to detect rotavirus-specific serum IgG, or IgA in the mice immunized intragastrically, independently of the use of the adjuvant. This was not the case for the groups immunized intranasally, with or without adjuvant, that presented rotavirus-specific serum IgG and IgA. However, the IgG titer was significantly higher in the group in which the adjuvant dmLT was included. Additionally, it is interesting to note that, despite the difference was not statistically significant, a greater signal was observed in the ELISA used for the detection of IgA in the group immunized with adjuvant and CWDP. On the other hand, before viral challenge, it was not possible to detect rotavirus-specific IgA in the feces of any of the evaluated groups by the conventional ELISA method used in this study.

The double mutant of the labile toxin (LT) of *E*. *coli* has two amino acid substitutions that reduce the enzyme activity, the enterotoxic activity *in vitro* and *in vivo*, but retains the adjuvant capacity (similar to mLT) [58]. Besides, it has been shown to be safe in mice and humans, at least when administered orally [58,59]. This modified toxin has been used successfully as an adjuvant in numerous studies and with different antigens and immunization routes [60–67].

Regarding the results obtained by intragastric immunization, studies carried out by Choi et al., 2002 [68] using recombinant VP6 of rotavirus produced in *E*. *coli* and different adjuvants showed that the intragastric route was less efficient than intranasal for the induction of a humoral immune response. Moreover, our results agree with several studies in which it is reported that the inclusion of the adjuvant dmLT promotes powerful immune responses [62,64,69].

However, it is worth noting that the CWDP without adjuvant were also able to induce a systemic humoral immune response after mucosal administration, in particular by the intranasal route. This result represents the first evidence that the VP6-CWDP generated in this work are capable of stimulating a systemic immune response after being administered by the intranasal route.

### Protection against RV infection

The adult mouse model represents a model of infection in which protection is evidenced by a reduction in viral excretion after the challenge. It has been described previously, in this model, that humoral immunity seems to be the main protection mechanism against rotavirus infection [70–73].

After challenging with the EC murine rotavirus strain, only the group administered intranasally with the VP6-CWDP and dmLT had a significant reduction in viral excretion (around 80%). Interestingly, before viral challenge, this group also presented a significantly higher anti-RV IgG titer respect to that immunized without the adjuvant.

Moreover, although the difference was not statically significant, a higher amount of serum anti-RV IgA was also found. When analyzing both IgG and serum IgA 10 days after the challenge, it was found that the group immunized intranasally with VP6-CWDP and dmLT (the one that had protection) significantly increased the IgA titer respect to the PBS group and the one immunized with the VP6-CWDP alone. A similar result was observed by Lappalainen et al., 2015 [74] that found a positive correlation between levels of serum IgA specific to VP6 post-challenge and protection against infection in mice.

Although external capsid proteins (VP4 and VP7) induce the production of neutralizing antibodies that confer protection, anti-VP6 IgA antibodies have been shown to be protective, possibly due to intracellular inhibition of viral replication [75–77].

Moreover, McNeal et al., 2006 [78] reported the intranasal immunization with a chimeric VP6 protein in the presence of the adjuvant mLT that managed to induce protection. In this study, using a BALB/c Jch -/- mice model, a 97% reduction in viral excretion (during the first seven days after challenge) was obtained although as expected, no specific IgA antibodies were detected in the intestinal lumen. On the contrary, high levels of VP6-specific IgA were detected in serum samples [78].

On the other hand, some studies have revealed the importance of cellular responses in the protection of infection using adjuvanted VP6 as immunogen [78,79]. Probably, in an immunocompetent mouse, both humoral and cellular responses have a synergic effect on the control of infection. However, this discussion is beyond the objective of the present paper.

In this work, a simple and inexpensive method is described for the generation of CWDP from *L*. *lactis* and their use as an effective immunogen. The antigen is produced by the same bacteria and the treatment to generate them keeps the proteins of *L*. *lactis* in their native state, guaranteeing the safety for its use because it comes from a GRAS organism. With this strategy, the production stages are simplified, and therefore the associated costs would be lower when compared to the BLP type strategy. Also, in the CWDP the antigen is covalently bound to the walls of *L*. *lactis*, which would substantially improve its stability. On the other hand, CWDP allowed concentrating the expressed protein and reducing the presence of most of the host-derived proteins, presenting advantages beyond the use of non-living *L*. *lactis*.

Future trials aimed to determine the mechanisms by which CWDP stimulate the immune system will be necessary. Furthermore, the performance of CWDP with other proteins derived from different pathogens and other administration routes could be evaluated.

## Supporting information

S1 Fig Rotavirus challengeot. y with or without dmLT presented both IgG and IgA specific antibodies against RV. described in th. Individual shedding curves obtained for each mouse within groups immunized with PBS, VP6-CWDP intranasally (**Vi**), VP6-CWDP intragastrically (**Vig**), VP6-CWDP + dmLT intranasally (**ViA**) or VP6-CWDP + dmLT intragastrically (**VigA**). Each symbol type represents the values obtained for the same mouse for each day.
(TIF)
