# Supplementary figures and images for: Rotavirus VP6 protein mucosally delivered by cell wall-derived particles from Lactococcus lactis induces protection against infection in a murine model
Source: PLoS One. 2018 Sep 7;13(9):e0203700. doi: 10.1371/journal.pone.0203700 (PMC6128627; doi:10.1371/journal.pone.0203700)

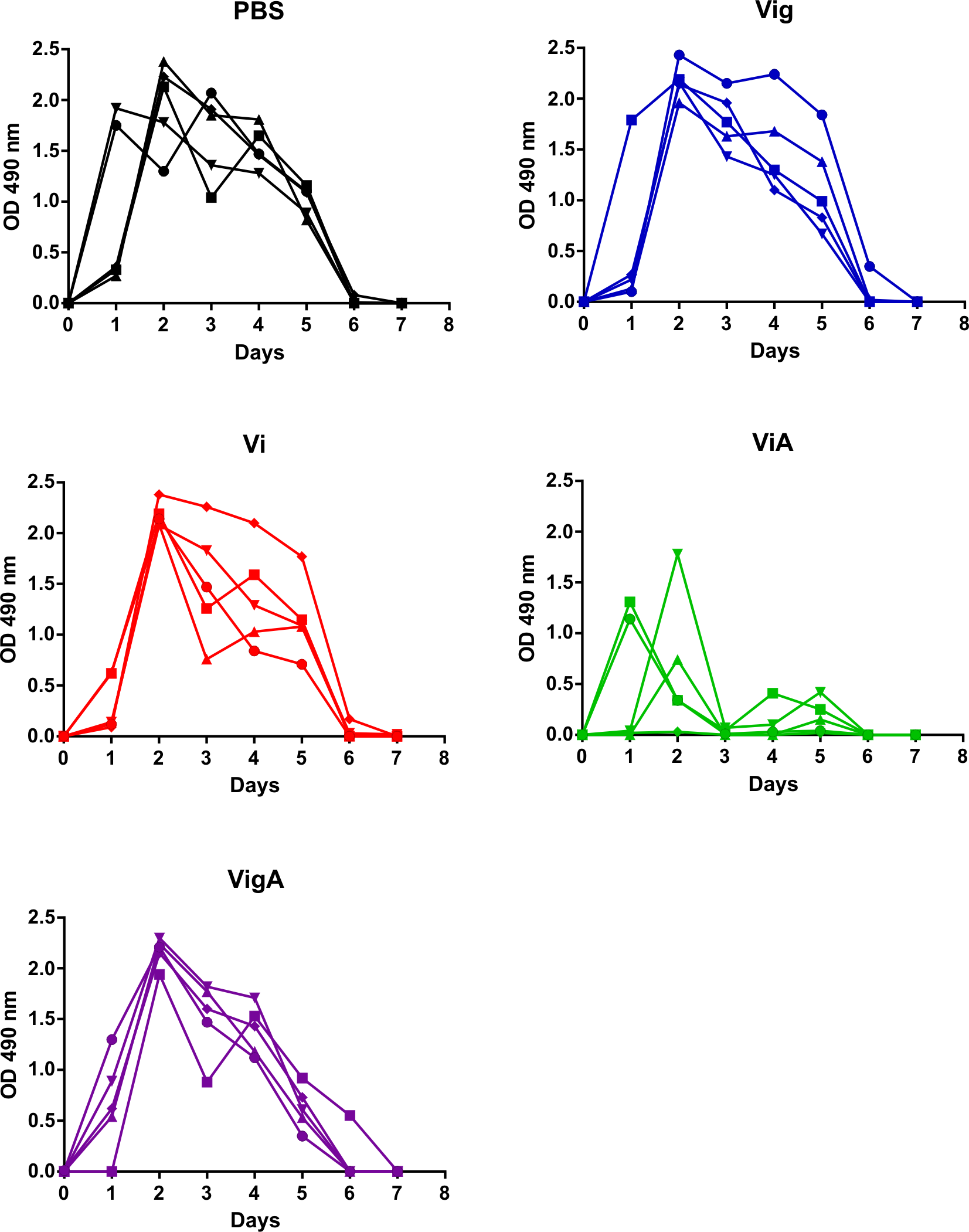

Supplement: S1 Fig — Individual shedding curves obtained for each mouse within groups immunized with PBS, VP6-CWDP intranasally (Vi), VP6-CWDP intragastrically (Vig), VP6-CWDP + dmLT intranasally (ViA) or VP6-CWDP + dmLT intragastrically (VigA). Each symbol type represents the values obtained for the same mouse for each day. (TIF) [file pone.0203700.s001.tif]
